# Accelerometer-Based Sedentary Time, Physical Activity, and Serum Metabolome in Young Men

**DOI:** 10.3390/metabo12080700

**Published:** 2022-07-27

**Authors:** Jani P. Vaara, Heikki Kyröläinen, Tommi Vasankari, Heikki Kainulainen, Jani Raitanen, Urho M Kujala

**Affiliations:** 1 Department of Leadership and Military Pedagogy, National Defence University, 00861 Helsinki, Finland; 2 Faculty of Sport and Health Science, University of Jyväskylä, 40014 Jyväskylän, Finland; heikki.kyrolainen@jyu.fi (H.K.); heikki.kainulainen@jyu.fi (H.K.); urho.m.kujala@fimnet.fi (U.M.K.); 3 UKK-Institute for Health Promotion Research, 33500 Tampere, Finland; tommi.vasankari@ukkinstituutti.fi (T.V.); jani.raitanen@ukkinstituutti.fi (J.R.); 4 Faculty of Medicine and Health Technology, Tampere University, 33100 Tampere, Finland; 5 Faculty of Social Sciences (Health Sciences), Tampere University, 33100 Tampere, Finland; 6 Special Services Unit, Finnish Institute for Health and Welfare, 00271 Helsinki, Finland

**Keywords:** objective physical activity, sedentary time, body composition, metabolomics, cardiovascular risk factors

## Abstract

Physical activity (PA) has been shown to associate with many health benefits but studies with metabolome-wide associations with PA are still lacking. Metabolome studies may deepen the mechanistic understanding of PA on the metabolic pathways related to health outcomes. The aim of the present study was to study the association of accelerometer based sedentary time (SB) and PA with metabolome measures. SB and PA were measured by a hip-worn accelerometer in 314 young adult men (age: mean 28, standard deviation 7 years). Metabolome was analyzed from fasting serum samples consisting of 66 metabolome measures (nuclear magnetic resonance-based metabolomics). The associations were analyzed using a single and compositional approach with regression analysis. The compositional analysis revealed that 4 metabolome variables were significantly (γ: 0.32–0.44, *p* ≤ 0.002), and 13 variables with a trend towards significance (*p* < 0.05), associated with SB with varying metabolic pathways. Trends towards significant associations (*p* < 0.05) were observed with 5 variables with moderate-to-vigorous and 1 variable with light intensity PA with varying metabolic pathways. The present study revealed possible mechanistic pathways relevant for the interaction between especially SB but also PA of moderate-to-vigorous intensity with ketone bodies and amino acid concentration related to exercised-induced energy production and lipid metabolism.

## 1. Introduction

Physical activity associates with many health benefits including cardiometabolic outcomes in adults and adolescents [1,2]. Often studies have concentrated on specific health outcomes, such as blood pressure or cholesterol concentrations. Recently, analyses of metabolome pattern have enabled to focus on metabolic outcomes and their pathways with broader aspects to elucidate deeper mechanistic insight. This is important since physical activity induces many physiological responses that together may be beneficial for overall health of an individual. Therefore, the assessment of metabolome-wide associations with physical activity may deepen our mechanistic understanding of the metabolic pathways related to health [3].

Although acute physical activity bouts have been shown to induce molecular responses in metabolism [4] and chronic physical activity to associate with health [5], only a few previous studies have investigated the relationships between physical activity and metabolomics. These previous metabolome studies have shown physical activity to associate with fatty acid profile, amino acids, carbohydrate metabolism, and inflammatory glycoproteins [6,7,8,9,10,11] with one study in adolescents showing no significant associations [12]. Besides these studies, there are fewer of those that have assessed metabolome- and accelerometer-based physical activity and sedentary time [7,10,13]. Accelerometer-based measures may allow more precise estimates of especially light intensity activity and sedentary time compared to the self-report method, known to include precision bias such as over-reporting or recall bias. Therefore, the purpose of the present study was to study the associations of accelerometer-based sedentary time and physical activity with targeted serum metabolome in a focused study sample consisting of young healthy adult men.

## 2. Results

The baseline characteristics including sedentary time and physical activity of the participants are presented in Table 1. Supplementary Table S1 describes the mean, standard deviation, and 95% confidence interval for serum metabolome outcomes.

The linear regression analysis is shown in Table 2 and the compositional analysis in Table 3 with the adjustments of age, education, smoking, alcohol, and nutrition. The level of statistical significance was found with a Bonferroni-corrected *p*-value of ≤0.002. The compositional analysis revealed that four metabolome variables (IDL concentration in TG, LDL concentration in TG, Acetoacetate, 3-hydroxybuturate) were significantly (*p* ≤ 0.002) positively associated with SB with varying metabolic pathways (Table 3). In addition, 13 (Very small VLDL, IDL, Large HDL, HDL diameter, ApoB, ApoB Apo A1 -ratio, HDL-C, HDL2-C, Unsaturation degree, Saturated FA ratio, Glycerol, Leucine, Valine) variables with a trend towards significance (*p* < 0.05) were positively associated with SB with varying metabolic pathways. The trend towards significant associations (*p* < 0.05) was also observed with five variables related to HDL metabolism (HDL-C, HDL2-C, ApoA1, Medium HDL, Large HDL) with moderate-to-vigorous physical activity, and one variable (lactate) with light intensity physical activity (*p* < 0.05) (Table 3). Further adjustments for either body fat percentage or aerobic fitness attenuated the associations mostly to non-significant (Tables S3 and S4). Moreover, Supplementary Table S2 demonstrates the associations between the accumulated physical activity intensities and serum metabolome biomarkers adjusted for age only.

Significant statistical differences were observed between individuals with high sedentary time and low MVPA compared to individuals with low sedentary time and high MVPA in saturated fatty acid ratio, and acetoacetate and 3-hydroxybuturate concentrations (*p* ≤ 0.001) (Table 4). In addition, there was a trend (*p* < 0.05) towards individuals with high sedentary time and low MVPA to exhibit higher concentrations of very small VLDL, IDL, large LDL, medium LDL and small LDL among lipoprotein particles, as well as higher apolipoprotein B and ApoB ApoA1 ratio compared to individuals with low sedentary time and high MVPA. Among triglycerides, the high sedentary time and low MVPA group exhibited a trend for higher triglyceride concentration in IDL and in LDL concentration, and among cholesterols, higher concentrations of IDL and LDL cholesterols compared to their counterparts (*p* < 0.05). Moreover, a trend for higher concentrations of saturated fatty acid ratio, glycerol, acetoacetate, 3-hydroxybuturate, and lower concentration of glutamine was observed in those with high sedentary time and low MVPA compared to their counterparts (*p* < 0.05).

## 3. Discussion

Among young healthy adult men, there were statistically significant and a trend towards significant associations of accelerometer-based sedentary time and physical activity with selected serum metabolome measures. Compositional analysis revealed that 4 metabolome variables were significantly associated with sedentary time and altogether 15 variables with a trend towards significant associations with varying metabolic pathways with sedentary time (13 variables) and moderate-to-vigorous physical activity (5 variables). The present study revealed possible mechanistic pathways relevant for the interaction between sedentary behavior, physical activity and ketone bodies as well as amino acid concentration both related to exercised-induced energy production and lipid metabolism. Moreover, selected lipoprotein, cholesterol, and fatty acid concentrations were trending towards significant associations with sedentary time and physical activity in young healthy adult men emphasizing the role of HDL metabolism.

Interestingly, one of the most consistent findings of the present study was that ketone bodies, acetoacetate and 3-hydroxybutrate were associated across all exposure variables, sedentary time and physical activity, in the linear regression analysis. We have previously observed similar associations in the same study sample [14], where both aerobic and maximal strength were associated with acetoacetate and 3-hydroxybutrate. Although only sedentary time was significantly associated with ketone bodies in the compositional analysis, these results may suggest that sedentary time, physical activity, and physical fitness may all be mechanistically related to fat catabolism. Interestingly, these associations remained even after adjustments for body fat or aerobic fitness for 3-hydroxybutrate and partly for acetoacetate. Previously, it has been postulated that BCAA degradation is linked with tricarboxylic acid cycle, intramyocellular lipid storage and oxidation enabling efficiency in lipid metabolism [15]. We also observed a trend towards significant associations in single regression models of sedentary time and physical activity with selected branch chain amino acids (BCAA), namely valine and leucine, but also with glutamine. Although only sedentary time was significantly associated with valine and leucine in the compositional analysis, the few previous study results support these findings [6,7]. These results may indicate that both reduced sedentary time and increased physical activity may have beneficial health-related associations with BCAA via their mechanistic pathways in physical-activity-induced energy production. In regards of this, the present study results emphasize the role of sedentary time. Possible mechanistic pathways may include the triggering effect of physical activity on BCAA oxidation in skeletal muscles and activation of enzymes related to protein degradation [16].

Previous studies with self-reported physical activity have shown associations with the metabolome in adults [6,8,9]; however, there are only a few accelerometer-based physical activity studies. These studies have shown selected associations between physical activity and metabolome. Bell et al. (2018) reported in children that total physical activity measured with accelerometer was significantly associated with fatty acid profile, namely cholesterol and triglycerides in HDL and VLDL particles, and glycoproteins acetyls with the strongest associations evident for higher intensity physical activity [10]. Although all associations were weak in magnitude, they were stronger for physical activity than for sedentary time. Another study on children [13] showed that accelerometer-based moderate-to-vigorous physical activity and sedentary time were associated with lipoprotein particle profile at the follow-up year after. In adults, accelerometer-based measurements of physical activity were inversely associated with selected amino acids and glucose metabolism among the metabolome profile [7]. Bell et al. (2018) reported in children that associations between metabolomics and MVPA tended to be greater than that of sedentary time [10]. In line with the present study, a recent meta-analysis [17] observed limited evidence for the association between light intensity physical activity and conventional cardiometabolic risk factors. In the present study, we found none of such associations in the compositional analysis, except a trend towards an inverse association between light intensity physical activity and lactate concentration, whereas moderate-to-vigorous physical activity was trending towards significant associations with five metabolome outcomes, all related to HDL metabolism.

Among the metabolome variables related to lipid metabolism with a trend towards significant associations with both sedentary time and moderate-to-vigorous physical activity, there were altogether seven variables directly related to HDL metabolism. This is in line with several previous studies, where an association between physical activity and HDL has been observed, e.g., [18,19]. The physiological mechanisms are not well elucidated, but it has been suggested in mouse models that HDL may enhance skeletal muscle cellular respiration [20]. Previous observational studies have, however, not shown consistent results for the association between accelerometer-based sedentary time and HDL cholesterol [21,22]. Unlike the present study, previous studies have only studied the total concentration of HDL and not, for example, the size of HDL particle sizes, which may reveal more clinically relevant information than total concentration alone.

We have previously shown that aerobic fitness, but not maximal strength, was associated with serum metabolome [14] in this same study sample. We further discussed that the metabolic pathways behind these associations could possibly relate to skeletal muscle metabolism [14]. Nevertheless, the associations observed between physical activity, sedentary time, and metabolome were fewer and consistently weaker in the present study. These results suggest that in the present study sample of young healthy men, aerobic fitness is a strong predictor of metabolites, which supports the earlier conclusions [23] and may emphasize that aerobic fitness, whether inherited or acquired, is more strongly associated with health outcomes than physical activity behavior, confirming some of the earlier findings [24,25,26].

Although it is well elucidated that an acute bout of physical activity and/or exercise induces acute changes in metabolism [4], the evidence of the effect of chronic physical activity and exercise seems less extensive and is limited to mainly observational studies including their limitations. A previous RCT study [27] reported modest changes in metabolome after longer-term (6 months) exercise training interventions. They did not observe significant changes between exercise and control groups in the metabolome pattern. Interestingly, significant changes were evident for all intervention groups when each individual served as his/her own control [27].

The present study has some limitations. The observed weak associations may partly be due to the young age of the participants. The relationships between physical activity and metabolome outcomes may not yet be strong in healthy children and young adults as metabolic and cardiovascular risk factors tend to accumulate over the human lifespan. Therefore, future studies are encouraged to study these associations with different samples, such as older and diseased individuals. Moreover, the accelerometer-based physical activity measurements, with at least a 4-day measurement period used in the present study, serves only a limited time of an individual`s life course and may therefore be, to some extent, inaccurate given that physical activity may fluctuate over time. Thus, future studies are warranted to use repeated measures of physical activity, even in accelerometer-based measurements. Finally, the cross-sectional study design does not allow for causal conclusions.

## 4. Materials and Methods

### 4.1. Participants

Participants were 776 young (age 26 ± 7 years) adult Finnish men, who were invited in the military refresher training. The information about the study plan for participants was sent to participants 5 months before the study conduction. The study was conducted in 7 different measurement sessions. Altogether, 1106 men were called up to the military refresher training and 823 participated in the training lasting 3–5 days. The most typical reasons for nonparticipation in the military refresher training were related to personal reasons, such as work-, study-, or health-related issues. Out of the 823 men, 776 participants participated in the study. Due to a high number of participants, we were able to give accelerometers only to 519 participants and analyze metabolome from 580 participants. Therefore, due to overlapping, the number of participants that had both accelerometer data (inclusion criteria as a minimum of 4 days with ≥10 h wear time per day) and serum metabolome analyzed consisted of 314 participants as the final study sample. The study protocol was explained in detail to the participants before they gave their written consent. The study was approved by the ethical committees of the University of Jyväskylä and the Central Finland Health Care District, and the Headquarters of the Finnish Defence Forces (AM5527).

### 4.2. Objectively Measured Physical Activity and Sedentary Time

A hip-worn triaxial accelerometer (Hookie AM 20, Traxmeet Ltd., Espoo, Finland) was used to assess physical activity and sedentary time. The accelerometer was attached to a flexible belt on the right side of the hip. Participants were given instructions to use the accelerometer for seven consecutive days during wake time. The acceleration data were collected at 100 Hz sampling rate and the raw accelerometer data were stored on a hard disk for further analysis. The mean amplitude deviation (MAD) values of the resultant acceleration of the three orthogonal acceleration components were determined in 6 s epochs. The MAD values have been found to be a valid indicator of incident energy expenditure during locomotion [28,29]. The MAD values were converted to METs for each epoch.

Physical activity was stratified into three intensity categories regarding METs: light physical activity 1.5–2.9 MET; moderate physical activity 3.0–5.9 MET; and vigorous physical activity > 6.0 MET [30]. Moreover, moderate and vigorous activities were combined to form moderate-to-vigorous physical activity (MVPA). Sedentary time was defined as the time spent in the seated/reclining/lying position without movement (<1.5 MET). The angle for the posture estimation algorithm was used to identify sedentary behavior (<1.5 MET). The assessment of the body posture was based on the fact that the Earth’s gravity vector is constant, and the body posture during walking is upright. The accelerometer orientation was used as a reference, and the angle for posture estimation was assessed from the concurrent accelerometer orientation relative to the reference vector [30,31].

### 4.3. Serum Metabolome

Circulating metabolomes (lipids, lipoproteins, and metabolites) were analyzed from blood samples using a high-throughput proton nuclear magnetic resonance spectroscopy metabolomics platform [32,33]. The platform represents a broad molecular signature of systemic metabolism with 153 measured metabolic traits [32,33] and quantifies various measures of lipoprotein metabolism, lipids, ketone bodies, and amino acids, as well as glycolysis and gluconeogenesis-related metabolites in absolute concentration units. In total, 66 metabolites or their ratios were selected for the present study similar to previous studies [6,14].

### 4.4. Demographic and Confounding Variables

Demographic and covariate variables were assessed with a questionnaire. Age, education, alcohol consumption, smoking habits (smoking, non-smoking, quit smoking) and nutritional habits (food frequency questions on vegetable, fruit, fish, chicken, and meat consumption) were assessed with the questionnaire. Body composition was measured with the bioelectrical impedance method (Inbody 720, Biospace Company, Seoul, Korea).

### 4.5. Statistics

Data was analyzed with Stata 15.1 for Windows. Descriptive statistics as means, standard deviations, 95% confidence intervals (CI) medians, and inter-quartile ranges (IQR) were calculated. Sedentary time, LPA, and MVPA variables were adjusted relative to total measurement time in all analyses. To study the associations of sedentary time and accumulated physical activity intensities, a similar approach was used previously [34], where firstly, linear regression models (Table 2) and secondly, compositional analysis (Table 3), were used (CoDaPack-software, v2.03.11). This approach takes into account that time is finite during the day and thus time spent in different behaviors are codependent. Linear regressions were used to calculate standardized regression coefficients (β) with 95% CIs *p*-level set at <0.002. First, we re-scaled the original time variables in different behaviors up to 1 (i.e., they varied between 0 and 1 and the sum of them was 1) and secondly, we made an isometric log-ratio (ILR) transformation for these proportions [33]. Finally, the new ILR-transformed variables were used as exposure variables in linear regression models where we used a similar adjustment (age, education, smoking, alcohol, and nutrition) as in our previous study [14] and further adjustments for body fat percentage (Table S3) and aerobic fitness (Table S4) were also calculated. In the present study, we used previous estimation of the multiple test correction for the interpretation of statistical significance in all the 66 studied metabolome measures. Previously, a principal component analysis was used to determine the minimum number of orthogonal linear components from a similar full metabolomics measures panel that explained 99% of the observed variance in a large data set [6]. The minimum number of orthogonal linear components was analyzed, and the highest number observed in the previously studied cohort [6] was 26 components, which we used as a consistent conservative estimate in all multiple testing interpretations using the Bonferroni method. A Bonferroni-corrected *p*-value of ≤0.002 was set as the level for statistical significance, which corresponds to a *p*-value of less than 0.05 [6]. Moreover, we assessed the low and high ends of sedentary time and MVPA combining their upper and lower tertiles to form a group with individuals with low sedentary time and high MVPA, and another group with individuals with low sedentary time and high MVPA. The comparisons between the groups were completed using independent *t*-tests and Mann–Whitney tests were further applied (Table 4).

## 5. Conclusions

The present study among young healthy adults showed, using compositional analysis, that 4 metabolome variables were significantly associated with sedentary time and altogether 15 variables with a trend towards significant associations with varying metabolic pathways with sedentary time (13 variables) and moderate-to-vigorous physical activity (5 variables). The present study, therefore, revealed possible mechanistic pathways relevant for interaction between, especially, sedentary time and moderate-to-vigorous physical activity but not light intensity physical activity with ketone bodies and amino acid concentration related to exercised-induced energy production, and lipid metabolism, emphasizing the role of HDL metabolism.

## Figures and Tables

**Table 1:** Demographic and baseline characteristics of the study population (*n* = 314).

Characteristics	Mean (Standard Deviation)	95% Confidence Interval
Age in years	28.2 (7.3)	27.4–29.0
Smokers, *n* (%)	75 (24.0)	19.6–29.0 *
**Sedentary time**		
Total sedentary time (min)	707.6 (127.3)	693.5–721.8
Total sedentary time (relative to accelerometer wear time, %)	77.6 (8.0)	76.8–78.5
**Physical activity (min)**		
Light Activity (1.5–3 MET) (min)	119.8 (47.1)	114.6–125.1
Moderate Activity (3–6 MET) (min)	77.8 (32.4)	74.2–81.4
Vigorous Activity (>6 MET) (min)	4.4 (6.3)	3.7–5.1
Moderate-to-Vigorous Activity (≥3 MET) (min)	82.1 (34.0)	78.4–85.9
**Physical activity relative to accelerometer wear time (%)**		
Light Activity (1.5–3 MET) (%)	13.2 (5.1)	12.7–13.8
Moderate Activity (3–6 MET) (%)	8.6 (3.6)	8.2–9.0
Vigorous Activity (>6 MET) (%)	0.48 (0.67)	0.41–0.56
Moderate-to-Vigorous Activity (≥3 MET) (%)	9.1 (3.8)	8.7–9.5

* The Wilson score method without continuity correction (Newcombe RG (1998) Two-sided confidence intervals for the single proportion: Comparison of seven methods. Statistics in Medicine, 17, 857–872.). MET = metabolic equivalent.

**Table 2:** Standardized single regression coefficients (β) of sedentary time and physical activity intensities with their 95% confidence intervals (CI) from age, education, smoking, alcohol, and nutrition-adjusted linear regression models for serum metabolome. Bonferroni-corrected *p*-value of ≤0.002 was set as the level for statistical significance, which corresponds to a *p*-value of less than 0.05.

	Sedentary Time		Light Intensity Physical Activity		Moderate-to-Vigorous Physical Activity	
Metabolome Measure	β (95% CI)	*p*-Value	β (95% CI)	*p*-Value	β (95% CI)	*p*-Value
**Lipoprotein particle concentration**						
ǂ Extremely large VLDL	0.07 (−0.03; 0.16)	0.191	−0.07 (−0.16; 0.03)	0.150	−0.05 (−0.16; 0.06)	0.345
ǂ Very large VLDL	0.09 (−0.02; 0.20)	0.111	−0.10 (−0.21; 0.02)	0.089	−0.06 (−0.16; 0.05)	0.285
* Large VLDL	0.06 (−0.06; 0.17)	0.342	−0.06 (−0.19; 0.06)	0.314	−0.06 (−0.17; 0.05)	0.296
ǂ Medium VLDL	0.08 (−0.04; 0.21)	0.196	−0.09 (−0.21; 0.04)	0.188	−0.08 (−0.21; 0.04)	0.196
Small VLDL	0.08 (−0.02; 0.18)	0.099	−0.09 (−0.19; 0.01)	0.085	−0.06 (−0.16; 0.04)	0.225
Very small VLDL	0.12 (0.02; 0.22)	0.021	−0.11 (−0.21; 0.00)	0.043	−0.11 (−0.21; −0.01)	0.028
IDL	0.11 (0.01; 0.21)	0.040	−0.09 (−0.19; 0.02)	0.108	−0.11 (−0.22; −0.01)	0.027
Large LDL	0.10 (−0.00; 0.21)	0.055	−0.08 (−0.19; 0.03)	0.140	−0.11 (−0.21; −0.01)	0.037
Medium LDL	0.10 (−0.00; 0.20)	0.059	−0.08 (−0.19; 0.03)	0.142	−0.11 (−0.21; −0.00)	0.042
Small LDL	0.10 (−0.01; 0.20)	0.072	−0.08 (−0.19; 0.03)	0.144	−0.10 (−0.20; 0.005)	0.062
Very large HDL	−0.09 (−0.20; 0.02)	0.128	0.06 (−0.06; 0.17)	0.333	0.11 (−0.00; 0.21)	0.055
Large HDL	−0.13 (−0.24; −0.02)	0.022	0.09 (−0.02; 0.21)	0.112	0.15 (0.04; 0.26)	0.006
Medium HDL	−0.08 (−0.19; 0.03)	0.147	0.06 (−0.05; 0.18)	0.298	0.09 (−0.02; 0.20)	0.094
Small HDL	0.04 (−0.07; 0.15)	0.487	−0.03 (−0.15; 0.08)	0.582	−0.04 (−0.15; 0.07)	0.462
**Lipoprotein particle size**						
VLDL diameter	0.05 (−0.06; 0.16)	0.401	−0.06 (−0.18; 0.06)	0.305	−0.02 (−0.14; 0.09)	0.669
LDL diameter	−0.02 (−0.13; 0.09)	0.759	0.04 (−0.07; 0.16)	0.443	−0.02 (−0.13; 0.09)	0.728
HDL diameter	−0.11 (−0.22; −0.01)	0.040	0.08 (−0.03; 0.20)	0.148	0.13 (0.02; 0.24)	0.016
**Apolipoproteins**						
ApoA1	−0.09 (−0.20; 0.02)	0.120	0.05 (−0.06; 0.17)	0.372	0.12 (0.01; 0.23)	0.037
ApoB	0.10 (−0.00; 0.21)	0.056	−0.10 (−0.20; 0.01)	0.081	−0.09 (−0.19; 0.01)	0.085
ApoB ApoA1 ratio	0.14 (0.03; 0.24)	0.012	−0.12 (−0.23; −0.01)	0.034	−0.13 (−0.24; −0.03)	0.012
**Triglycerides**						
ǂ Total triglycerides	0.12 (−0.00; 0.24)	0.055	−0.10 (−0.22; 0.02)	0.090	−0.05 (−0.17; 0.07)	0.446
ǂ VLDL TG	0.07 (−0.06; 0.19)	0.300	−0.07 (−0.19; 0.06)	0.308	−0.06 (−0.18; 0.07)	0.390
IDL TG	0.18 (0.08; 0.27)	<0.001 *	−0.16 (−0.25; −0.06)	0.001 *	−0.16 (−0.26; −0.07)	<0.001 *
LDL TG	0.15 (0.06; 0.25)	0.001 *	−0.14 (−0.23; −0.04)	0.005	−0.15 (−0.24; −0.05)	0.002 *
HDL TG	0.03 (−0.07; 0.12)	0.573	−0.05 (−0.15; 0.05)	0.339	0.00 (−0.09; 0.10)	0.953
**Cholesterol**						
Total cholesterol	0.07 (−0.04; 0.17)	0.207	−0.06 (−0.17; 0.05)	0.269	−0.06 (−0.17; 0.04)	0.230
VLDL-C	0.08 (−0.03; 0.19)	0.137	−0.09 (−0.19; 0.02)	0.120	−0.06 (−0.16; 0.05)	0.275
IDL-C	0.10 (−0.01; 0.20)	0.064	−0.08 (−0.19; 0.03)	0.160	−0.11 (−0.21; −0.00)	0.041
LDL-C	0.10 (−0.01; 0.20)	0.074	−0.07 (−0.18; 0.03)	0.179	−0.10 (−0.21; −0.00)	0.047
HDL-C	−0.11 (−0.22; 0.01)	0.064	0.08 (−0.04; 0.19)	0.194	0.12 (0.01; 0.23)	0.029
HDL2-C	−0.11 (−0.22; −0.00)	0.046	0.08 (−0.03; 0.20)	0.149	0.13 (0.02; 0.24)	0.022
HDL3-C	0.02 (−0.09; 0.13)	0.731	−0.04 (−0.15; 0.07)	0.517	0.01 (−0.10; 0.11)	0.905
Esterified cholesterol	0.07 (−0.04; 0.17)	0.217	−0.06 (−0.17; 0.05)	0.283	−0.06 (−0.17; 0.04)	0.237
Free cholesterol	0.07 (−0.03; 0.17)	0.187	−0.06 (−0.17; 0.04)	0.239	−0.06 (−0.17; 0.04)	0.219
**Fatty acids**						
Total fatty acids	0.07 (−0.04; 0.17)	0.207	−0.08 (−0.19; 0.03)	0.142	−0.04 (−0.14; 0.06)	0.462
Unsaturation degree	−0.12 (−0.23; −0.01)	0.028	0.12 (0.01; 0.23)	0.031	0.10 (−0.01; 0.20)	0.074
Omega-3 FA	0.01 (−0.09; 0.12)	0.831	−0.02 (−0.13; 0.09)	0.732	0.00 (−0.10; 0.10)	0.999
Omega-3 FA ratio	−0.08 (−0.18; 0.03)	0.168	0.07 (−0.04; 0.19)	0.193	0.06 (−0.04; 0.17)	0.233
Docosahexaenoic acid	0.04 (−0.07; 0.14)	0.466	−0.05 (−0.15; 0.06)	0.379	−0.02 (−0.12; 0.08)	0.700
Polyunsaturated FA	0.05 (−0.06; 0.15)	0.372	−0.06 (−0.16; 0.05)	0.311	−0.03 (−0.13; 0.07)	0.580
Omega-6 FA	0.05 (−0.05; 0.16)	0.320	−0.06 (−0.17; 0.05)	0.267	−0.03 (−0.14; 0.07)	0.522
Omega-6 FA ratio	−0.09 (−0.20; 0.02)	0.126	0.10 (−0.02; 0.21)	0.093	0.06 (−0.05; 0.16)	0.307
Linoleic acid	0.05 (−0.06; 0.15)	0.372	−0.06 (−0.17; 0.05)	0.290	−0.03 (−0.13; 0.08)	0.620
Saturated FA	0.08 (−0.02; 0.19)	0.124	−0.09 (−0.20; 0.01)	0.088	−0.05 (−0.16; 0.05)	0.315
Saturated FA ratio	0.13 (0.02; 0.25)	0.025	−0.12 (−0.24; 0.00)	0.051	−0.12 (−0.24; −0.01)	0.032
Monounsaturated FA	0.06 (−0.04; 0.17)	0.235	−0.08 (−0.19; 0.03)	0.136	−0.03 (−0.13; 0.08)	0.577
**Metabolic substrates**						
Glucose	−0.09 (−0.19; 0.01)	0.086	0.11 (0.01; 0.21)	0.035	0.04 (−0.06; 0.14)	0.390
Glycerol	0.11 (0.00; 0.22)	0.041	−0.08 (−0.19; 0.03)	0.175	−0.13 (−0.24; −0.03)	0.012
Acetoacetate	−0.02 (−0.13; 0.09)	0.724	0.02 (−0.09; 0.14)	0.674	0.01 (−0.10; 0.12)	0.847
ǂ 3-hydroxybuturate	0.13 (0.05; 0.21)	0.002 *	−0.11 (−0.20; −0.03)	0.011	−0.13 (−0.21; −0.05)	0.001 *
ǂ Acetate	0.16 (0.05; 0.26)	0.003	−0.16 (−0.27; −0.05)	0.003	−0.15 (−0.25; −0.05)	0.003
Citrate	0.00 (−0.11; 0.11)	0.996	−0.02 (−0.14; 0.10)	0.725	0.03 (−0.09; 0.14)	0.658
Lactate	0.08 (−0.03; 0.18)	0.148	−0.10 (−0.20; 0.01)	0.067	−0.03 (−0.13; 0.07)	0.514
Pyruvate	0.04 (−0.06; 0.14)	0.403	0.00 (−0.10; 0.11)	0.957	−0.09 (−0.19; 0.01)	0.070
**Amino acids**						
Isoleucine (BCAA)	0.10 (−0.01; 0.22)	0.081	−0.11 (−0.23; 0.01)	0.062	−0.07 (−0.18; 0.04)	0.217
Leucine (BCAA)	0.13 (0.02; 0.24)	0.024	−0.14 (−0.26; −0.02)	0.019	−0.10 (−0.21; 0.02)	0.092
Valine	0.16 (0.05; 0.27)	0.004	−0.16 (−0.27; −0.05)	0.006	−0.13 (−0.24; −0.02)	0.018
Alanine	0.02 (−0.09; 0.14)	0.674	−0.05 (−0.17; 0.06)	0.379	0.01 (−0.10; 0.12)	0.798
Glutamine	−0.19 (−0.29; −0.09)	<0.001	0.20 (0.10; 0.30)	<0.001 *	0.13 (0.04; 0.23)	0.008
Glycine	−0.07 (−0.18; 0.04)	0.185	0.08 (−0.03; 0.19)	0.167	0.05 (−0.05; 0.16)	0.330
Histidine	0.03 (−0.09; 0.14)	0.664	−0.06 (−0.17; 0.06)	0.351	0.02 (−0.09; 0.13)	0.767
Phenylalanine	0.04 (−0.07; 0.16)	0.444	−0.05 (−0.17; 0.06)	0.378	−0.03 (−0.14; 0.08)	0.647
Tyrosine	−0.06 (−0.17; 0.06)	0.317	0.06 (−0.06; 0.17)	0.357	0.05 (−0.06; 0.16)	0.372
**Miscellaneous**						
Glycoproteins	0.07 (−0.04; 0.18)	0.193	−0.05 (−0.16; 0.06)	0.342	−0.08 (−0.19; 0.03)	0.137
Creatinine	0.06 (−0.05; 0.17)	0.290	−0.08 (−0.20; 0.03)	0.165	−0.02 (−0.13; 0.09)	0.681
Albumin	0.02 (−0.08; 0.11)	0.762	−0.05 (−0.15; 0.05)	0.345	0.03 (−0.07; 0.13)	0.560

ǂ Quantile regression models applied. * statistically significant, *p* ≤ 0.002.

**Table 3:** Compositional analysis with standardized regression coefficients (β) of sedentary time and physical activity intensities for age, education, smoking, alcohol, nutrition-adjusted regression models for serum metabolome. Bonferroni-corrected *p*-value of ≤0.002 was set as the level for statistical significance, which corresponds to a *p*-value of less than 0.05.

	Sedentary Time		Light Intensity Physical Activity		Moderate-to-Vigorous Physical Activity	
Metabolome Measure	γ	*p*-Value	γ	*p*-Value	γ	*p*-Value
**Lipoprotein particle concentration**						
ǂ Extremely large VLDL	0.091	0.458	−0.284	0.157	0.194	0.289
ǂ Very large VLDL	0.130	0.354	−0.351	0.127	0.221	0.290
ǂ Large VLDL	0.111	0.440	−0.235	0.316	0.124	0.559
ǂ Medium VLDL	0.177	0.251	−0.141	0.576	−0.036	0.875
Small VLDL	0.221	0.082	−0.265	0.201	0.044	0.817
Very small VLDL	0.305	0.018	−0.142	0.499	−0.163	0.395
IDL	0.270	0.041	−0.010	0.961	−0.259	0.186
Large LDL	0.252	0.059	0.002	0.994	−0.253	0.200
Medium LDL	0.248	0.063	−0.010	0.965	−0.238	0.229
Small LDL	0.237	0.076	−0.049	0.823	−0.188	0.342
Very large HDL	−0.164	0.228	−0.135	0.541	0.299	0.138
Large HDL	−0.332	0.018	−0.116	0.610	0.448	0.031
Medium HDL	−0.249	0.079	−0.187	0.419	0.436	0.039
Small HDL	0.076	0.594	−0.174	0.456	0.098	0.643
**Lipoprotein particle size**						
VLDL diameter	0.114	0.431	−0.238	0.314	0.124	0.563
LDL diameter	−0.037	0.794	0.344	0.136	−0.307	0.143
HDL diameter	−0.300	0.031	−0.075	0.743	0.374	0.071
**Apolipoproteins**						
ApoA1	−0.243	0.088	−0.237	0.310	0.480	0.024
ApoB	0.265	0.048	−0.141	0.519	−0.124	0.533
ApoB ApoA1 ratio	0.358	0.008	−0.059	0.787	−0.299	0.134
**Triglycerides**						
ǂ Total triglycerides	0.245	0.105	−0.430	0.083	0.185	0.411
ǂ VLDL TG	0.133	0.383	−0.248	0.320	0.115	0.612
IDL TG	0.444	<0.001 *	−0.203	0.293	−0.240	0.173
LDL TG	0.386	0.001 *	−0.166	0.391	−0.219	0.215
HDL TG	0.088	0.474	−0.284	0.156	0.196	0.283
**Cholesterol**						
Total cholesterol	0.164	0.222	−0.086	0.695	−0.078	0.697
VLDL-C	0.219	0.104	−0.218	0.323	−0.001	0.997
IDL-C	0.244	0.068	0.018	0.933	−0.262	0.187
LDL-C	0.235	0.080	0.020	0.927	−0.255	0.202
HDL-C	−0.289	0.042	−0.134	0.564	0.424	0.046
HDL2-C	−0.309	0.029	−0.111	0.630	0.420	0.046
HDL3-C	0.045	0.746	−0.284	0.211	0.239	0.246
Esterified cholesterol	0.160	0.235	−0.085	0.221	−0.075	0.201
Free cholesterol	0.172	0.196	−0.088	0.688	−0.084	0.670
**Fatty acids**						
Total fatty acids	0.179	0.183	−0.282	0.200	0.103	0.606
Unsaturation degree	−0.295	0.033	0.269	0.235	0.026	0.898
Omega-3 FA	0.040	0.764	−0.114	0.602	0.074	0.710
Omega-3 FA ratio	−0.178	0.198	0.119	0.598	0.059	0.776
Docosahexaenoic acid	0.106	0.423	−0.199	0.359	0.093	0.638
Polyunsaturated FA	0.124	0.354	−0.201	0.358	0.077	0.697
Omega-6 FA	0.137	0.308	−0.213	0.332	0.076	0.702
Omega-6 FA ratio	−0.212	0.131	0.286	0.214	−0.074	0.725
Linoleic acid	0.125	0.355	−0.236	0.287	0.111	0.581
Saturated FA	0.221	0.101	−0.292	0.187	0.070	0.725
Saturated FA ratio	0.352	0.016	−0.092	0.702	−0.261	0.231
Monounsaturated FA	0.170	0.210	−0.319	0.152	0.149	0.462
**Metabolic substrates**						
Glucose	−0.241	0.060	0.409	0.051	−0.168	0.377
Glycerol	0.299	0.030	0.083	0.712	−0.382	0.061
ǂ Acetoacetate	0.443	0.001 *	−0.175	0.437	−0.268	0.192
ǂ 3-hydroxybuturate	0.319	0.002 *	−0.029	0.863	−0.290	0.055
Acetate	−0.048	0.737	0.043	0.854	0.005	0.982
Citrate	0.047	0.748	−0.101	0.672	0.054	0.802
Lactate	0.208	0.109	−0.457	0.032	0.248	0.200
Pyruvate	−0.080	0.536	0.264	0.212	−0.184	0.338
**Amino acids**						
Isoleucine (BCAA)	0.262	0.073	−0.274	0.252	0.011	0.959
Leucine (BCAA)	0.339	0.019	−0.354	0.135	0.015	0.946
Valine	0.396	0.005	−0.361	0.117	−0.035	0.867
Alanine	0.061	0.669	−0.227	0.334	0.166	0.437
Glutamine	−0.059	0.649	0.149	0.485	−0.090	0.644
Glycine	−0.159	0.253	0.250	0.271	−0.091	0.659
Histidine	0.101	0.483	−0.412	0.082	0.310	0.148
Phenylalanine	0.109	0.447	−0.126	0.592	0.017	0.938
Tyrosine	−0.134	0.359	0.121	0.614	0.013	0.951
**Miscellaneous**						
Glycoproteins	0.167	0.222	−0.06	0.942	−0.151	0.460
Creatinine	0.201	0.159	−0.352	0.132	0.151	0.476
Albumin	0.071	0.572	−0.400	0.052	0.329	0.079

ǂ Quantile regression models applied. * statistically significant, *p* ≤ 0.002.

**Table 4:** Comparison (mean with standard deviation (SD) and median with interquartile range (IQR)) between a group with high sedentary time and low moderate-to-vigorous physical activity (MVPA) (*n* = 77), and a group with low sedentary time and high MVPA (*n* = 86).

	High Sedentary Time and Low MVPA		Low Sedentary Time and High MVPA		
Metabolome Measure	Mean (SD)	Median (IQR)	Mean (SD)	Median (IQR)	*p*-Value
**Lipoprotein particle concentration**					
Extremely large VLDL	1.0 × 10^−10^ (9.8 × 10^−11^)	7.7 × 10^−11^ (8.6 × 10^−11^)	8.6 × 10^−11^ (1.1 × 10^−10^)	6.1 × 10^−11^ (1.1 × 10^−10^)	0.097 ^1^
Very large VLDL	4.7 × 10^−10^ (5.8 × 10^−10^)	2.7 × 10^−10^ (6.3 × 10^−10^)	4.2 × 10^−10^ (6.1 × 10^−10^)	2.1 × 10^−10^ (5.8 × 10^−10^)	0.332 ^1^
Large VLDL	3.6 × 10^−09^ (3.3 × 10^−09^)	2.4 × 10^−09^ (3.3 × 10^−09^)	3.2 × 10^−09^ (3.5 × 10^−09^)	2.3 × 10^−09^ (3.0 × 10^−09^)	0.259 ^1^
Medium VLDL	1.5 × 10^−08^ (8.4 × 10^−09^)	1.1 × 10^−08^ (9.0 × 10^−09^)	1.3 × 10^−08^ (8.8 × 10^−09^)	1.1 × 10^−08^ (7.8 × 10^−09^)	0.251 ^1^
Small VLDL	2.6 × 10^−08^ (9.8 × 10^−09^)	2.4 × 10^−08^ (1.3 × 10^−08^)	2.4 × 10^−08^ (9.9 × 10^−09^)	2.2 × 10^−08^ (5.4 × 10^−08^)	0.174 ^1^
Very small VLDL	3.6 × 10^−08^ (8.5 × 10^−09^)	3.5 × 10^−08^ (1.3 × 10^−08^)	3.3 × 10^−08^ (7.7 × 10^−09^)	3.3 × 10^−08^ (9.6 × 10^−09^)	0.017 ^2^
IDL	9.9 × 10^−08^ (2.4 × 10^−08^)	9.5 × 10^−08^ (3.7 × 10^−08^)	8.9 × 10^−08^ (2.1 × 10^−08^)	8.9 × 10^−08^ (2.6 × 10^−08^)	0.009 ^2^
Large LDL	1.6 × 10^−07^ (4.3 × 10^−08^)	1.6 × 10^−07^ (6.2 × 10^−08^)	1.5 × 10^−07^ (3.7 × 10^−08^)	1.4 × 10^−07^ (5.0 × 10^−08^)	0.013 ^2^
Medium LDL	1.3 × 10^−07^ (3.7 × 10^−08^)	1.3 × 10^−07^ (5.1 × 10^−08^)	1.2 × 10^−07^ (3.2 × 10^−08^)	1.2 × 10^−07^ (4.3 × 10^−08^)	0.017 ^2^
Small LDL	1.5 × 10^−07^ (4.1 × 10^−08^)	1.5 × 10^−07^ (5.6 × 10^−08^)	1.4 × 10^−07^ (3.6 × 10^−08^)	1.4 × 10^−07^ (4.7 × 10^−08^)	0.023 ^2^
Very large HDL	3.7 × 10^−07^ (1.4 × 10^−07^)	3.5 × 10^−07^ (2.0 × 10^−07^)	3.9 × 10^−07^ (1.4 × 10^−07^)	3.8 × 10^−07^ (2.1 × 10^−07^)	0.331 ^2^
Large HDL	7.5 × 10^−07^ (3.4 × 10^−07^)	7.5 × 10^−07^ (5.2 × 10^−07^)	8.6 × 10^−07^ (3.6 × 10^−07^)	8.3 × 10^−07^ (5.7 × 10^−07^)	0.051 ^2^
Medium HDL	1.6 × 10^−06^ (2.4 × 10^−07^)	1.5 × 10^−06^ (3.5 × 10^−07^)	1.6 × 10^−06^ (2.4 × 10^−07^)	1.6 × 10^−06^ (2.9 × 10^−07^)	0.316 ^2^
Small HDL	4.6 × 10^−06^ (4.0 × 10^−07^)	4.6 × 10^−06^ (6.3 × 10^−07^)	4.5 × 10^−06^ (3.5 × 10^−07^)	4.5 × 10^−06^ (4.4 × 10^−07^)	0.348 ^2^
**Lipoprotein particle size**					
VLDL diameter	36.4 (1.2)	36.2 (1.4)	36.3 (1.3)	36.2 (1.7)	0.639 ^2^
LDL diameter	23.6 (0.1)	23.6 (0.1)	23.6 (0.1)	23.6 (0.1)	0.912 ^2^
HDL diameter	9.8 (0.2)	9.8 (0.4)	9.8 (0.2)	9.8 (0.3)	0.121 ^2^
**Apolipoproteins**					
ApoA1	1.31 (0.12)	1.27 (0.15)	1.33 (0.13)	1.34 (0.17)	0.343 ^2^
ApoB	0.82 (0.19)	0.80 (0.25)	0.76 (0.17)	0.75 (0.23)	0.031 ^2^
ApoB ApoA1 ratio	0.63 (0.15)	0.60 (0.19)	0.57 (0.13)	0.56 (0.16)	0.015 ^2^
**Triglycerides**					
Total triglycerides	1.07 (0.48)	0.92 (0.53)	0.99 (0.51)	0.87 (0.44)	0.158 ^1^
VLDL TG	0.73 (0.42)	0.57 (0.45)	0.67 (0.44)	0.57 (0.38)	0.232 ^1^
IDL TG	0.092 (0.022)	0.090 (0.033)	0.084 (0.023)	0.080 (0.022)	0.007 ^1^
LDL TG	0.14 (0.037)	0.13 (0.053)	0.12 (0.037)	0.12 (0.040)	0.010 ^1^
HDL TG	0.12 (0.029)	0.11 (0.037)	0.11 (0.031)	0.10 (0.032)	0.259 ^1^
**Cholesterol**					
Total cholesterol	3.92 (0.79)	3.85 (1.14)	3.69 (0.73)	3.65 (0.90)	0.058 ^2^
VLDL-C	0.61 (0.23)	0.58 (0.29)	0.56 (0.23)	0.52 (0.27)	0.075 ^1^
IDL-C	0.64 (0.17)	0.62 (0.24)	0.58 (0.14)	0.57 (0.18)	0.012 ^2^
LDL-C	1.51 (0.45)	1.46 (0.63)	1.35 (0.39)	1.34 (0.53)	0.018 ^2^
HDL-C	1.16 (0.22)	1.13 (0.27)	1.21 (0.24)	1.20 (0.35)	0.154 ^2^
HDL2-C	0.67 (0.21)	0.66 (0.26)	0.72 (0.22)	0.71 (0.32)	0.117 ^2^
HDL3-C	0.49 (0.02)	0.49 (0.03)	0.49 (0.02)	0.49 (0.03)	0.543 ^2^
Esterified cholesterol	2.80 (0.57)	2.76 (0.81)	2.64 (0.52)	2.60 (0.63)	0.060 ^2^
Free cholesterol	1.12 (0.22)	1.11 (0.33)	1.06 (0.21)	1.05 (0.25)	0.057 ^2^
**Fatty acids**					
Total fatty acids	9.41 (1.93)	9.26 (2.62)	8.95 (1.95)	8.75 (2.40)	0.137 ^2^
Unsaturation degree	1.22 (0.05)	1.23 (0.06)	1.24 (0.05)	1.23 (0.05)	0.060 ^2^
Omega-3 FA	0.36 (0.10)	0.34 (0.13)	0.34 (0.10)	0.33 (0.12)	0.442 ^2^
Omega-3 FA ratio	3.76 (0.57)	3.65 (0.76)	3.81 (0.59)	3.73 (0.80)	0.534 ^2^
Docosahexaenoic acid	0.096 (0.033)	0.091 (0.043)	0.090 (0.033)	0.085 (0.038)	0.277 ^2^
Polyunsaturated FA	3.72 (0.64)	3.71 (0.84)	3.59 (0.61)	3.53 (0.74)	0.199 ^2^
Omega-6 FA	3.36 (0.55)	3.34 (0.74)	3.25 (0.52)	3.18 (0.69)	0.176 ^2^
Omega-6 FA ratio	36.1 (2.6)	36.5 (3.4)	36.7 (2.7)	37.3 (3.2)	0.138 ^2^
Linoleic acid	2.89 (0.47)	2.89 (0.7)	2.81 (0.44)	2.76 (0.59)	0.266 ^2^
Saturated FA	3.23 (0.68)	3.16 (0.95)	3.02 (0.69)	2.96 (0.85)	0.064 ^2^
Saturated FA ratio	34.27 (1.00)	34.39 (1.24)	33.74 (1.06)	33.85 (1.30)	0.001 ^2,^*
Monounsaturated FA	2.47 (0.70)	2.34 (0.90)	2.34 (0.73)	2.25 (0.86)	0.132 ^1^
**Metabolic substrates**					
Glucose	4.00 (0.38)	3.96 (0.49)	4.04 (0.36)	4.04 (0.42)	0.356 ^1^
Glycerol	0.072 (0.023)	0.066 (0.027)	0.063 (0.019)	0.061 (0.022)	0.008 ^1^
Acetoacetate	0.119 (0.080)	0.095 (0.100)	0.081 (0.053)	0.059 (0.048)	<0.001 ^1,^*
3-hydroxybuturate	0.21 (0.13)	0.17 (0.15)	0.16 (0.10)	0.13 (0.07)	<0.001 ^1,^*
Acetate	0.044 (0.009)	0.043 (0.014)	0.043 (0.010)	0.042 (0.013)	0.532 ^2^
Citrate	0.107 (0.011)	0.107 (0.016)	0.107 (0.012)	0.105 (0.017)	0.749 ^2^
Lactate	0.92 (0.24)	0.89 (0.31)	0.89 (0.23)	0.81 (0.32)	0.263 ^1^
Pyruvate	0.079 (0.017)	0.077 (0.018)	0.075 (0.013)	0.075 (0.018)	0.150 ^2^
**Amino acids**					
Isoleucine (BCAA)	0.058 (0.012)	0.057 (0.015)	0.056 (0.015)	0.054 (0.017)	0.096 ^1^
Leucine (BCAA)	0.085 (0.013)	0.084 (0.017)	0.081 (0.015)	0.079 (0.019)	0.139 ^2^
Valine	0.184 (0.023)	0.184 (0.039)	0.178 (0.027)	0.181 (0.034)	0.132 ^2^
Alanine	0.365 (0.052)	0.361 (0.070)	0.359 (0.042)	0.347 (0.058)	0.445 ^2^
Glutamine	0.502 (0.059)	0.498 (0.078)	0.537 (0.085)	0.529 (0.103)	0.005 ^1^
Glycine	0.229 (0.027)	0.224 (0.032)	0.233 (0.027)	0.232 (0.034)	0.282 ^1^
Histidine	0.069 (0.006)	0.069 (0.009)	0.069 (0.007)	0.069 (0.009)	0.737 ^1^
Phenylalanine	0.068 (0.009)	0.066 (0.010)	0.068 (0.007)	0.067 (0.007)	0.741 ^2^
Tyrosine	0.051 (0.009)	0.05 (0.013)	0.052 (0.009)	0.053 (0.011)	0.496 ^2^
**Miscellaneous**					
Glycoproteins	1.25 (0.18)	1.21 (0.23)	1.23 (0.17)	1.20 (0.17)	0.566 ^1^
Creatinine	0.071 (0.009)	0.070 (0.012)	0.069 (0.008)	0.069 (0.011)	0.159 ^2^
Albumin	0.089 (0.005)	0.089 (0.007)	0.089 (0.005)	0.089 (0.007)	0.572 ^2^

^1^ Mann–Whitney test, ^2^ independent-samples *t*-test. * statistically significant, *p* ≤ 0.002.

## Data Availability

The Finnish Defence Forces own and manage the data, which are available for researchers who meet the criteria for access to confidential data. For data access, contact Dr. Jani Vaara, Department of Leadership and Military Pedagogy, National Defence University, 00861 Helsinki, Finland; e-mail: jani.vaara@mil.fi; tel.: +358-299530432.

## Supplementary Materials

The following supporting information can be downloaded at: https://www.mdpi.com/article/10.3390/metabo12080700/s1, Table S1: The descriptive (mean, standard deviation (sd), and 95% confidence interval (CI)) characteristics for serum metabolome measures; Table S2: Standardized single regression coefficients (β) of sedentary time and physical activity intensities with their 95% confidence intervals (CI) from age-adjusted linear regression models for serum metabolome; Table S3: Standardized single regression coefficients (β) of sedentary time and physical activity intensities with their 95% confidence intervals (CI) from age, education, smoking, alcohol, nutrition, and body fat percentage-adjusted linear regression models for serum metabolome; Table S4: Standardized single regression coefficients (β) of sedentary time and physical activity intensities with their 95% confidence intervals (CI) from age, education, smoking, alcohol, nutrition, and aerobic-fitness-adjusted linear regression models for serum metabolome.
