# Supplementary material for: Accelerometer-Based Sedentary Time, Physical Activity, and Serum Metabolome in Young Men
Source: Metabolites. 2022 Jul 27;12(8):700. doi: 10.3390/metabo12080700 (PMC9414649; doi:10.3390/metabo12080700)
Supplement: Supplementary file 1 [file metabolites-12-00700-s001.zip › metabolites-1774573-supplementary files_07_08_2022_JVa-edited.pdf]

Table S1: The descriptive (mean, standard deviation (sd) and 95% confidence interval (CI)) characteristics for serum metabolome measures

Metabolome measure	Mean (sd)	95% CI	Metabolome measure	Mean (sd)	95% CI
**Lipoprotein particle concentration**			**Apolipoproteins**		
Extremely large VLDL	9.2×10^{-11} (9.3×10^{-11})	$8.2\times 10^{-11} – 1.0\times 10^{-10}$	ApoA1	1.32 (0.13)	1.31 – 1.33
Very large VLDL	4.4×10^{-10} (5.3×10^{-10})	$3.8\times 10^{-10} – 5.0\times 10^{-10}$	ApoB	0.78 (0.18)	0.76 – 0.80
Large VLDL	3.4×10^{-09} (3.1×10^{-09})	$3.0\times 10^{-09} – 3.7\times 10^{-09}$	ApoB ApoA1 ratio	0.59 (0.14)	0.58 – 0.61
Medium VLDL	1.4×10^{-08} (7.9×10^{-09})	$1.3\times 10^{-08} – 1.5\times 10^{-08}$			
Small VLDL	2.5×10^{-08} (9.3×10^{-09})	$2.4\times 10^{-08} – 2.6\times 10^{-08}$	**Cholesterol**		
Very small VLDL	3.4×10^{-08} (8.4×10^{-09})	$3.3\times 10^{-08} – 3.5\times 10^{-08}$	Total cholesterol	3.77 (0.79)	3.69 – 3.86
IDL	9.3×10^{-08} (2.4×10^{-08})	$9.0\times 10^{-08} – 9.5\times 10^{-08}$	VLDL-C a	0.58 (0.23)	0.55 – 0.60
Large LDL	1.5×10^{-07} (4.2×10^{-08})	$1.5\times 10^{-07} – 1.6\times 10^{-07}$	IDL-C	0.60 (0.17)	0.58 – 0.62
Medium LDL	1.2×10^{-07} (3.7×10^{-08})	$1.2\times 10^{-07} – 1.3\times 10^{-07}$	LDL-C	1.41 (0.45)	1.36 – 1.46
Small LDL	1.4×10^{-07} (4.1×10^{-08})	$1.4\times 10^{-07} – 1.5\times 10^{-07}$	HDL-C	1.19 (0.23)	1.16 – 1.21
Very large HDL	3.7×10^{-07} (1.4×10^{-07})	$3.5\times 10^{-07} – 3.8\times 10^{-07}$	HDL2-C	0.70 (0.22)	0.68 – 0.72
Large HDL	8.0×10^{-07} (3.5×10^{-07})	$7.7\times 10^{-07} – 8.4\times 10^{-07}$	HDL3-C	0.488 (0.023)	0.486 – 0.491
Medium HDL	1.6×10^{-06} (2.5×10^{-07})	$1.6\times 10^{-06} – 1.6\times 10^{-06}$	Esterified cholesterol	2.69 (0.57)	2.63 – 2.76
Small HDL	4.6×10^{-06} (3.7×10^{-07})	$4.5\times 10^{-06} – 4.6\times 10^{-06}$	Free cholesterol	1.08 (0.22)	1.05 – 1.10
**Fatty acids**			**Metabolic substrates**		
Total fatty acids	9.1 (1.9)	8.9 – 9.3	Glucose	4.02 (0.35)	3.98 – 4.06
Unsaturation degree	1.23 (0.04)	1.22 – 1.23	Glycerola	0.067 (0.021)	0.065 – 0.070
Omega-3 FA	0.34 (0.10)	0.33 – 0.35	Acetoacetatea	0.098 (0.072)	0.090 – 0.106
Omega-3 FA ratio	3.72 (0.57)	3.65 – 3.78	3-hydroxybuturate	0.18 (0.13)	0.17 – 0.20
Docosahexaenoic acid	0.090 (0.032)	0.086 – 0.093	Acetate	0.044 (0.010)	0.042 – 0.045
Polyunsaturated FA	3.63 (0.65)	3.56 – 3.70	Citrate	0.106 (0.011)	0.105 – 0.107
Omega-6 FA	3.29 (0.56)	3.23 – 3.35	Lactatea	0.90 (0.25)	0.88 – 0.93
Omega-6 FA ratio	36.4 (2.4)	36.1 – 36.6	Pyruvate	0.078 (0.015)	0.076 – 0.080
Linoleic acid	2.84 (0.48)	2.79 – 2.90			
Saturated FAa	3.10 (0.67)	3.03 – 3.18	**Amino acids**		
Saturated FA ratio	34.0 (1.0)	33.9 – 34.1	Isoleucine (BCAA)	0.057 (0.013)	0.056 – 0.058
Monounsaturated FA	2.39 (0.68)	2.32 – 2.47	Leucine (BCAA)	0.083 (0.013)	0.081 – 0.084
			Valine	0.182 (0.025)	0.179 – 0.184
**Lipoprotein particle size**			Alanine	0.359 (0.047)	0.354 – 0.364

VLDL diameter	36.4 (1.2)	36.3 – 36.5	Glutamine	0.520 (0.068)	0.512 – 0.528
LDL diameter	23.6 (0.1)	23.6 – 23.6	Glycinea	0.231 (0.028)	0.228 – 0.234
HDL diameter	9.82 (0.2)	9.79 – 9.84	Histidine	0.068 (0.007)	0.068 – 0.069
			Phenylalaninea	0.068 (0.008)	0.067 – 0.069
**Triglycerides**			Tyrosine	0.051 (0.009)	0.051 – 0.052
Total triglycerides	1.02 (0.45)	0.97 – 1.07			
VLDL TG	0.70 (0.39)	0.65 – 0.74	**Miscellanous**		
IDL TG	0.086 (0.022)	0.084 – 0.088	Glycoproteins	1.24 (0.17)	1.23 – 1.26
LDL TG	0.13 (0.04)	0.12 – 0.13	Creatinine	0.069 (0.008)	0.068 – 0.070
HDL TG	0.11 (0.03)	0.11 – 0.12	Albumin	0.089 (0.005)	0.088 – 0.089

Table S2: Standardized single regression coefficients (β) of sedentary time and physical activity intensities with their 95% confidence intervals (CI) from age-adjusted linear regression models for serum metabolome

Metabolome measure	Sedentary time		Light intensity physical activity		Moderate physical activity		Vigorous physical activity		Moderate-to-vigorous physical activity	
	β (95% CI)	p-value	β (95% CI)	p-value	β (95% CI)	p-value	β (95% CI)	p-value	β (95% CI)	p-value
**Lipoprotein particle concentration**										
ǂExtremely large VLDL (mmol/l)	0.052 (0.050; 0.153)	0.318	-0.045 (-0.146; 0.055)	0.373	-0.042 (-0.150; 0.066)	0.444	-0.114 (-0.210; -0.020)	0.018	-0.047 (-0.152; 0.059)	0.383
ǂVery large VLDL (mmol/l)	0.014 (-0.100; 0.128)	0.808	-0.018 (-0.134; 0.100)	0.757	-0.007; (-0.121; 0.107)	0.148	-0.079 (-0.186; 0.028)	0.148	-0.009 (-0.122; 0.105)	0.881
ǂLarge VLDL (mmol/l)	0.011 (-0.101; 0.122)	0.849	-0.010 (-0.117; 0.097)	0.854	0.005 (-0.101; 0.110)	0.930	-0.120 (-0.224; -0.016)	0.024	-0.011 (-0.122; 0.101)	0.826
ǂMedium VLDL (mmol/l)	0.038 (-0.088; 0.164)	0.550	-0.031 (-0.156; 0.094)	0.626	-0.036 (-0.161; 0.088)	0.567	-0.109 (-0.225; 0.007)	0.066	-0.053 (-0.180; 0.074)	0.413
Small VLDL (mmol/l)	0.073 (-0.025; 0.170)	0.143	-0.067 (-0.164; 0.031)	0.179	-0.051 (-0.149; 0.047)	0.306	-0.088 (-0.185; 0.009)	0.077	-0.065 (-0.162; 0.033)	0.194
Very small VLDL (mmol/l)	0.112 (0.014; 0.211)	0.026	-0.091 (-0.190; 0.007)	0.071	-0.110 (-0.209; -0.011)	0.029	-0.057 (-0.157; 0.041)	0.255	-0.116 (-0.215; -0.017)	0.022
IDL (mmol/l)	0.114 (0.012; 0.215)	0.028	-0.089 (-0.190; 0.012)	0.084	-0.119 (-0.220; -0.017)	0.021	-0.039 (-0.140; 0.063)	0.457	-0.120 (-0.222; -0.020)	0.019
Large LDL (mmol/l)	0.106 (0.005; 0.208)	0.040	-0.083 (-0.184; 0.019)	0.112	-0.113 (-0.215; -0.011)	0.029	-0.035 (-0.137; 0.067)	0.499	-0.115 (-0.216; -0.013)	0.027

Medium LDL (mmol/l)	0.102 (0.001; 0.204)	0.048	-0.079 (-0.180; 0.023)	0.129	-0.109 (-0.210; -0.007)	0.036	-0.038 (-0.140; 0.064)	0.462	-0.111 (-0.212; -0.010)	0.032
Small LDL (mmol/l)	0.097 (-0.005; 0.198)	0.062	-0.077 (-0.179; 0.024)	0.136	-0.100 (-0.201; 0.002)	0.055	-0.033 (-0.135; 0.069)	0.524	-0.101 (-0.203; 0.001)	0.050
Very large HDL (mmol/l)	-0.026 (-0.134; 0.083)	0.640	-0.016 (-0.124; 0.092)	0.775	0.053 (-0.056; 0.161)	0.342	0.054 (0.038; 0.251)	0.008	0.077 (-0.032; 0.185)	0.166
Large HDL (mmol/l)	-0.101 (-0.210; 0.008)	0.069	0.049 (-0.060; 0.159)	0.375	0.120 (0.011; 0.229)	0.031	0.184 (0.077; 0.292)	0.001 *	0.148 (0.039; 0.256)	0.008
Medium HDL (mmol/l)	-0.080 (-0.189; 0.029)	0.151	0.055 (-0.054; 0.164)	0.322	0.076 (-0.033; 0.186)	0.169	0.122 (0.014; 0.231)	0.027	0.095 (-0.014; 0.204)	0.087
Small HDL (mmol/l)	0.014 (-0.097; 0.124)	0.805	0.006 (-0.105; 0.116)	0.919	-0.029 (-0.139; 0.082)	0.612	-0.056 (-0.166; 0.055)	0.322	-0.037 (-0.148; 0.073)	0.508
**Lipoprotein particle size**										
VLDL diameter (nm)	0.027 (-0.084; 0.139	0.629	-0.027 (-0.138; 0.084)	0.634	-0.003 (-0.114; 0.109)	0.961	-0.107 (-0.218; 0.003)	0.057	-0.022 (-0.133; 0.090)	0.701
LDL diameter (nm)	0.005 (-0.104; 0.114)	0.927	0.013 (-0.095; 0.122)	0.812	-.033 (-0.141; 0.076)	0.555	0.016 (-0.093; 0.124)	0.778	-0.028 (-0.137; 0.080)	0.606
HDL diameter (nm)	-0.082 (-0.192; 0.027)	0.141	0.034 (-0.076; 0.144)	0.543	0.101 (-0.008; 0.211)	0.070	0.179 (0.071; 0.288)	0.001 *	0.129 (0.020; 0.238)	0.021
**Apolipoproteins**										
ApoA1 (g/l)	-0.069 (-0.180; 0.043)	0.227	0.023 (-0.089; 0.134)	0.691	0.091 (-0.021; 0.202)	0.110	0.159 (0.049; 0.269)	0.005	0.115 (0.004; 0.226)	0.042
ApoB (g/l)	0.098 (-0.003; 0.200)	0.058	-0.085 (-0.187; 0.017)	0.100	-0.085 (-0.187; 0.017)	0.101	-0.069 (-0.171; 0.033)	0.182	-0.094 (-0.196; 0.008)	0.070
ApoB ApoA1 ratio	0.123 (0.021; 0.225)	0.018	-0.094 (-0.196; 0.008)	0.072	-0.116 (-0.218; -0.014)	0.026	-0.132 (-0.234; -0.030)	0.026	-0.135 (-0.237; -0.033)	0.010
**Triglycerides**										

ǂTotal triglycerides (mmol/l)	0.054 (-0.072; 0.180)	0.399	-0.046 (-0.175; 0.083)	0.488	-0.055 (-0.185; 0.076)	0.090	-0.105 (-0.226; 0.017)	0.090	-0.074 (-0.204; 0.056)	0.263
ǂVLDL TG (mmol/l)	0.037 (-.081; 0.154)	0.542	-0.025 (-0.144; 0.093)	0.673	-0.030 (-0.150; 0.088)	0.612	-0.129 (-0.245; -0.012)	0.030	-0.060 (-0.181; 0.060)	0.325
IDL TG (mmol/l)	0.161 (0.069; 0.252)	0.001 *	-0.125 (-0.217; -0.033)	0.008	-0.164 (-0.256; -0.072)	0.001 *	-0.086 (-0.179 ; 0.006)	0.068	-0.173 (-0.264; -0.081)	<0.001 *
LDL TG (mmol/l)	0.144 (0.052; 0.236)	0.002 *	-0.113 (-0.205; -0.021)	0.017	-0.145 (-0.237; -0.053)	0.002 *	-0.078 (-0.170; 0.0142)	0.097	-0.153 (-0.245; -0.061)	0.001 *
HDL TG (mmol/l)	0.030 (-0.066; 0.125)	0.541	-0.042 (-0.137; 0.053)	0.388	0.004 (-0.091; 0.100)	0.927	-0.059 (-0.154; 0.035)	0.219	-0.006 (-0.102; 0.089)	0.896
**Cholesterol**										
Total cholesterol (mmol/l)	0.077 (-0.027; 0.180)	0.145	-0.070 (-0.173; 0.034)	0.185	-0.073 (-0.176; 0.031)	0.169	0.005 (-0.099; 0.108)	0.932	-0.069 (-0.172; 0.035)	0.192
VLDL-C (mmol/l)	0.071 (-0.031; 0.174)	0.173	-0.067 (-0.170; 0.035)	0.198	-0.048 (-0.151; 0.054)	0.355	-0.080 (-0.182; 0.023)	0.127	-0.061 (-0.163; 0.042)	0.246
IDL-C (mmol/l)	0.108 (.006; 0.211)	0.038	-0.086 (-0.189; 0.017)	0.100	-0.113 (-0.216; -0.011)	0.031	-0.031 (-0.134; 0.072)	0.558	-0.114 (-0.216; -0.011)	0.029
LDL-C (mmol/l)	0.101 (-0.001; 0.204)	0.052	-0.078 (-0.181; 0.024)	0.136	-0.109 (-0.212; -0.007)	0.036	-0.029 (-0.132; 0.073)	0.574	-0.110 (-0.213; -0.007)	0.035
HDL-C (mmol/l)	-0.084 (-0.194; 0.027)	0.137	0.041 (-0.069; 0.152)	0.460	0.094 (-0.016; 0.205)	0.094	0.176 (0.067; 0.284)	0.002 *	0.122 (0.012; 0.232)	0.030
HDL2-C (mmol/l)	-0.091 (-0.201; 0.019)	0.103	0.049 (-0.061; 0.159)	0.382	0.100 (-0.010; 0.209)	0.074	0.178 (0.070; 0.286)	0.001 *	0.127 (0.018; 0.237)	0.022
HDL3-C (mmol/l)	0.029 (-0.077; 0.134)	0.593	-0.048 (-0.154; 0.057)	0.364	-0.006 (-0.112; 0.099)	0.899	0.063 (-0.042; 0.168)	0.242	0.005 (-0.101; 0.110)	0.931
Esterified cholesterol (mmol/l)	0.076 (-0.028; 0.179)	0.151	-0.069 (-0.172; 0.035)	0.193	-0.072 (-0.176; 0.031)	0.171	0.007 (-0.097; 0.111)	0.890	-0.068 (-0.172; 0.036)	0.197
Free cholesterol (mmol/l)	0.078 (-0.024; 0.181)	0.135	-0.071 (-0.174; 0.031)	0.172	-0.072 (-0.175; 0.031)	0.168	-0.003 (-0.106; 0.100)	0.960	-0.070 (-0.172; 0.033)	0.183
**Fatty acids**										
Total fatty acids (mmol/l)	0.063 (-0.040; 0.166)	0.228	-0.069 (-0.171; 0.034)	0.187	-0.037 (-0.140; 0.066)	0.481	-0.031 (-0.134; 0.072)	0.551	-0.041 (-0.143; 0.062)	0.435

Unsaturation degree	-0.088 (-0.198; 0.022)	0.115	0.066 (-0.044; 0.176)	0.237	0.070 (-0.040; 0.180)	0.209	0.172 (0.063; 0.280)	0.002 *	0.098 (-0.012; 0.207)	0.081
Omega-3 FA (mmol/l)	0.027 (-0.076; 0.130)	0.608	-0.038 (-0.141; 0.064)	0.463	-0.012 (-0.115; 0.091)	0.814	0.037 (-0.066; 0.140)	0.480	-0.005 (-0.108; 0.098)	0.920
Omega-3 FA ratio	-0.037 (-0.146; 0.073)	0.509	0.014 (-0.096; 0.123)	0.806	0.041 (-0.069; 0.150)	0.466	0.117 (0.009; 0.226)	0.035	0.060 (-0.050; 0.169)	0.284
Docosahexaenoic acid (mmol/l)	0.056 (-0.047; 0.159)	0.283	-0.069 (-0.171; 0.034)	0.191	-0.035 (-0.139; 0.068)	0.501	0.038 (-0.066; 0.141)	0.472	-0.027 (-0.130; 0.076)	0.604
Polyunsaturated FA (mmol/l)	0.055 (-0.048; 0.157)	0.295	-0.062 (-0.165; 0.041)	0.236	-0.037 (-0.140; 0.066)	0.480	0.015 (-0.088; 0.119)	0.770	-0.033 (-0.136; 0.070)	0.531
Omega-6 FA (mmol/l)	0.059 (-0.044; 0.162)	0.263	-0.065 (-0.168; 0.038)	0.215	-0.041 (-0.144; 0.062)	0.438	0.011 (-0.092; 0.114)	0.833	-0.037 (-0.140; 0.066)	0.480
Omega-6 FA ratio	-0.062 (-0.170; 0.047)	0.263	0.056 (-0.053; 0.164)	0.314	0.035 (-0.074; 0.144)	0.524	0.127 (0.019; 0.235)	0.022	0.056 (-0.053; 0.165)	0.310
Linoleic acid (mmol/l)	0.052 (-0.052; 0.156)	0.326	-0.060 (-0.164; 0.043)	0.254	-0.032 (-0.136; 0.072)	0.543	0.011 (-0.094; 0.115)	0.842	-0.029 (-0.133; 0.075)	0.584
Saturated FA (mmol/l)	0.080 (-0.023; 0.183)	0.127	-0.084 (-0.187; 0.019)	0.108	-0.052 (-0.155; 0.051)	0.319	-0.035 (-0.138; 0.068)	0.507	-0.056 (-0.159; 0.047)	0.284
Saturated FA ratio	0.144 (0.033; 0.254)	0.011	-0.128 (-0.239; -0.017)	0.023	-0.131 (-0.242; -0.020)	0.021	-0.036 (-0.148; 0.077)	0.533	-0.132 (-0.243; -0.021)	0.020
Monounsaturated FA (mmol/l)	0.048 (-0.056; 0.152)	0.369	-0.053 (-0.158; 0.051)	0.313	-0.018 (-0.122; 0.087)	0.740	-0.069 (-0.174; 0.035)	0.194	-0.029 (-0.133; 0.075)	0.584
**Metabolic substrates**										
Glucose (mmol/l)	-0.097 (-0.195; 0.001)	0.052	0.115 (0.017; 0.212)	0.021	0.063 (-0.035; 0.162)	0.208	-0.051 (-0.149; 0.047)	0.308	0.051 (-0.047; 0.150)	0.306
Glycerola (mmol/l)	0.107 (0.002; 0.212)	0.046	-0.064 (-0.169; 0.042)	0.236	-0.127 (-0.232; -0.022)	0.017	-0.104 (-0.209; 0.001)	0.051	-0.141 (-0.245; -0.036)	0.009
Acetoacetatea (mmol/l)	0.190 (0.089; 0.291)	<0.001 *	-0.144 (-0.248; -0.041)	0.006	-0.144 (-0.247; -0.040)	0.007	-0.090 (-0.190; 0.010)	0.079	-0.148 (-0.254; -0.041)	0.007

ǂ3-hydroxybuturate (mmol/l)	0.137 (0.060; 0.213)	0.001 *	-0.117 (-0.189-0.044)	0.002 *	-0.128 (-0.203; -0.053)	0.001 *	-0.031 (-0.103; 0.042)	0.409	-0.130 (-0.206; -0.054)	0.001 *
ǂ Acetate (mmol/l)	0.008 (-0.101; 0.117)	0.878	-0.012 (-0.121; 0.097)	0.829	0.009 (-0.099; 0.117)	0.867	-0.060 (-0.169; 0.048)	0.273	-0.002 (-0.111; 0.107)	0.973
Citrate (mmol/l)	-0.021 (-0.132; 0.092)	0.724	0.005 (-0.106; 0.117)	0.922	0.038 (-0.073; 0.150)	0.500	-0.009 (-0.121; 0.102)	0.873	0.035 (-0.077; 0.147)	0.536
Lactatea (mmol/l)	0.057 (-0.043; 0.157)	0.260	-0.070 (-0.170; 0.029)	0.168	-0.032 (-0.132; 0.068)	0.529	0.021 (-0.079; 0.121)	0.679	-0.027 (-0.127; 0.073)	0.596
Pyruvate (mmol/l)	0.018 (-0.079; 0.117)	0.709	0.038 (-0.060; 0.136)	0.448	-0.073 (-0.170; 0.025)	0.145	-0.116 (-0.213; -0.019)	0.019	-0.090 (-0.188; 0.007)	0.069
**Amino acids**										
Isoleucine (BCAA) (mmol/l)	0.085 (-0.026; 0.196)	0.133	-0.086 (-0.197; 0.025)	0.127	-0.046 (-0.157; 0.066)	0.420	-0.115 (-0.226; -0.005)	0.041	-0.064 (-0.176; 0.047)	0.256
Leucine (BCAA) (mmol/l)	0.111 (-0.001; 0.221)	0.051	-0.109 (-0.220; 0.002)	0.055	-0.068 (-0.179; 0.044)	0.234	-0.132 (-0.243; -0.022)	0.019	-0.088 (-0.200; 0.023)	0.119
Valine (mmol/l)	0.154 (0.045; 0.263)	0.006	-0.147 (-0.256; -0.038)	0.008	-0.121 (-0.231; -0.012)	0.030	-0.130 (-0.239; -0.020)	0.195	-0.154 (-0.263; -0.045)	0.020
Alanine (mmol/l)	-0.002 (-0.112; 0.109)	0.978	-0.015 (-0.125; 0.095)	0.784	0.036 (-0.074; 0.146)	0.516	-0.061 (-0.171; 0.049)	0.273	0.024 (-0.086; 0.134)	0.669
Glutamine (mmol/l)	-0.194 (-0.289; -0.098)	<0.001 *	0.202 (0.107; 0.297)	<0.001 *	0.138 (0.041; 0.234)	0.005	0.035 (-0.062; 0.132)	0.483	0.138 (0.041; 0.235)	0.005
Glycinea (mmol/l)	-0.084 (-0.189; 0.021)	0.116	0.087 (-0.018; 0.191)	0.105	0.077 (-0.027; 0.183)	0.147	-0.072 (-0.176; 0.033)	0.180	0.062 (-0.043; 0.167)	0.249
Histidine (mmol/l)	-0.005 (-0.116; 0.105)	0.926	-0.013 (-0.123; 0.098)	0.818	0.035 (-0.076; 0.145)	0.540	-0.025 (-0.136; 0.085)	0.656	0.029 (-0.082; 0.139)	0.611
Phenylalaninea (mmol/l)	-0.007 (-0.119; 0.104)	0.899	0.020 (-0.092; 0.132)	0.725	0.015 (-0.097; 0.127)	0.793	-0.146 (-0.256; -0.035)	0.010	-0.012 (-0.123; 0.100)	0.838
Tyrosine (mmol/l)	-0.073 (-0.184; 0.039)	0.201	0.069 (-0.042; 0.180)	0.224	0.082 (-0.030; 0.193)	0.151	-0.097 (-0.208; 0.014)	0.087	0.061 (-0.051; 0.173)	0.283
**Miscellaneous**										
Glycoproteins (mmol/l)	0.037 (-0.069; 0.143)	0.491	-0.002 (-0.108; 0.103)	0.965	-0.048 (-0.155; 0.060)	0.383	-0.171 (-0.275; -0.068)	0.001 *	-0.077 (-0.184; 0.030)	0.157
Creatinine (g/l)	0.087 (-0.023; 0.197)	0.119	-0.117 (-0.226; -0.008)	0.119	-0.029 (-0.139; 0.081)	0.606	0.002 (-0.108; 0.112)	0.606	-0.027 (-0.138; 0.083)	0.625
Albumin (g/l)	0.016 (-0.081; 0.114)	0.740	-0.050 (-0.147; 0.046)	0.307	0.021 (-0.076; 0.118)	0.667	0.072 (-0.025; 0.169)	0.144	0.033 (-0.064; 0.130)	0.501

ǂ Quantile regression models applied,* $p \leq 0.002$.

Table S3: Standardized single regression coefficients (β) of sedentary time and physical activity intensities with their 95% confidence intervals (CI) from age, education, smoking, alcohol, nutrition and body fat percentage-adjusted linear regression models for serum metabolome

Metabolome measure	Sedentary time		Light intensity physical activity		Moderate-to-vigorous phsical activity	
	β (95% CI)	p-value	β (95% CI)	p-value	β (95% CI)	p-value
**Lipoprotein particle concentration**						
ǂ Extremely large VLDL	-0.01 (-0.11; 0.09)	0.816	-0.01 (-0.10; 0.09)	0.896	0.03 (-0.07; 0.13)	0.532
ǂ Very large VLDL	0.02 (-0.09; 0.13)	0.776	-0.02 (-0.12; 0.09)	0.720	-0.002 (-0.10; 0.10)	0.970
ǂ Large VLDL	0.02 (-0.08; 0.12)	0.667	-0.02 (-0.13; 0.08)	0.679	-0.02 (-0.12; 0.08)	0.685
ǂ Medium VLDL	0.01 (-0.09; 0.11)	0.854	-0.03 (-0.14; 0.08)	0.570	0.03 (-0.08; 0.13)	0.620
Small VLDL	-0.02 (-0.11; 0.07)	0.652	0.004 (-0.09; 0.10)	0.930	0.04 (-0.05; 0.13)	0.411
Very small VLDL	0.03 (-0.07; 0.13)	0.551	-0.03 (-0.13; 0.07)	0.598	-0.03 (-0.12; 0.07)	0.580
IDL	0.03 (-0.07; 0.13)	0.563	-0.02 (-0.12; 0.09)	0.765	-0.04 (-0.14; 0.06)	0.418
Large LDL	0.02 (-0.08; 0.12)	0.672	-0.01 (-0.11; 0.10)	0.877	-0.03 (-0.13; 0.07)	0.500
Medium LDL	0.02 (-0.09; 0.12)	0.743	-0.005 (-0.11; 0.10)	0.926	-0.03 (-0.13; 0.07)	0.576
Small LDL	0.01 (-0.09; 0.12)	0.800	-0.01 (-0.11; 0.10)	0.920	-0.02 (-0.12; 0.08)	0.692
Very large HDL	0.01 (-0.10; 0.11)	0.906	-0.03 (-0.14; 0.08)	0.602	0.02 (-0.08; 0.13)	0.669
Large HDL	-0.02 (-0.12; 0.08)	0.723	-0.01 (-0.11; 0.10)	0.855	0.05 (-0.05; 0.15)	0.332
Medium HDL	-0.05 (-0.16; 0.07)	0.435	0.03 (-0.09; 0.14)	0.658	0.06 (-0.05; 0.17)	0.297
Small HDL	-0.04 (-0.15; 0.07)	0.517	0.04 (-0.08; 0.15)	0.520	0.03 (-0.08; 0.14)	0.610
**Lipoprotein particle size**						
VLDL diameter	-0.05 (-0.16; 0.06)	0.395	0.03 (-0.09; 0.14)	0.657	0.06 (-0.04; 0.17)	0.235
LDL diameter	0.03 (-0.08; 0.14)	0.609	0.004 (-0.11; 0.12)	0.948	-0.06 (-0.18; 0.05)	0.254
HDL diameter	0.003 (-0.10; 0.10)	0.949	-0.03 (-0.13; 0.08)	0.632	0.02 (-0.07; 0.12)	0.627
**Apolipoproteins**						
ApoA1	-0.05 (-0.17; 0.07)	0.405	0.01 (-0.10; 0.13)	0.819	0.08 (-0.03; 0.20)	0.153
ApoB	-0.003 (-0.10; 0.10)	0.953	-0.002 (-0.10; 0.10)	0.975	0.01 (-0.09; 0.10)	0.872
ApoB ApoA1 ratio	0.02 (-0.08; 0.11)	0.742	-0.01 (-0.11; 0.09)	0.861	-0.02 (-0.12; 0.07)	0.649
**Triglycerides**						
ǂ Total triglycerides	-0.0004 (-0.11; 0.11)	0.995	-0.01 (-0.12; 0.11)	0.910	0.04 (-0.07; 0.14)	0.487
ǂ VLDL TG	0.002 (-0.11; 0.12)	0.972	-0.03 (-0.14; 0.09)	0.657	0.04 (-0.07; 0.15)	0.486
IDL TG	0.10 (0.01; 0.19)	0.037	-0.08 (-0.18; 0.01)	0.072	-0.09 (-0.18; -0.002)	0.046
LDL TG	0.08 (-0.02; 0.17)	0.102	-0.06 (-0.16; 0.03)	0.165	-0.07 (-0.16; 0.02)	0.113
HDL TG	-0.05 (-0.14; 0.05)	0.311	0.02 (-0.08; 0.11)	0.687	0.07 (-0.02; 0.16)	0.115

**Cholesterol**						
Total cholesterol	0.003 (-0.10; 0.11)	0.959	-0.003 (-0.11; 0.11)	0.956	-0.002 (-0.11; 0.10)	0.971
VLDL-C	-0.03 (-0.12; 0.07)	0.616	0.01 (-0.09; 0.11)	0.874	0.04 (-0.06; 0.14)	0.405
IDL-C	0.02 (-0.08; 0.13)	0.658	-0.01 (-0.11; 0.10)	0.873	-0.04 (-0.14; 0.06)	0.478
LDL-C	0.02 (-0.09; 0.12)	0.735	-0.004 (-0.11; 0.10)	0.948	-0.03 (-0.13; 0.07)	0.538
HDL-C	-0.02 (-0.13; 0.09)	0.758	-0.01 (-0.12; 0.11)	0.925	0.04 (-0.07; 0.15)	0.448
HDL2-C	-0.02 (-0.13; 0.09)	0.739	-0.003 (-0.11; 0.11)	0.953	0.04 (-0.06; 0.15)	0.444
HDL3-C	0.002 (-0.11; 0.12)	0.971	-0.02 (-0.14; 0.09)	0.699	0.02 (-0.09; 0.13)	0.673
Esterified cholesterol	0.002 (-0.10; 0.11)	0.967	-0.002 (-0.11; 0.11)	0.970	-0.002 (-0.11; 0.10)	0.970
Free cholesterol	0.004 (-0.10; 0.11)	0.939	-0.01 (-0.11; 0.10)	0.923	-0.002 (-0.10; 0.10)	0.972
**Fatty acids**						
Total fatty acids	-0.03 (-0.13; 0.07)	0.591	0.004 (-0.10; 0.11)	0.945	0.05 (-0.05; 0.15)	0.306
Unsaturation degree	-0.02 (-0.12; 0.09)	0.757	0.03 (-0.08; 0.13)	0.600	-0.002 (-0.10; 0.10)	0.972
Omega-3 FA	-0.05 (-0.15; 0.06)	0.376	0.03 (-0.07; 0.14)	0.551	0.06 (-0.05; 0.16)	0.288
Omega-3 FA ratio	-0.05 (-0.17; 0.06)	0.352	0.05 (-0.06; 0.17)	0.372	0.04 (-0.07; 0.15)	0.440
Docosahexaenoic acid	-0.0004 (-0.11; 0.11)	0.994	-0.01 (-0.12; 0.09)	0.793	0.02 (-0.08; 0.12)	0.724
Polyunsaturated FA	-0.02 (-0.13; 0.08)	0.645	0.01 (-0.10; 0.11)	0.881	0.04 (-0.06; 0.14)	0.448
Omega-6 FA	-0.02 (-0.13; 0.09)	0.710	0.004 (-0.10; 0.11)	0.948	0.04 (-0.07; 0.14)	0.494
Omega-6 FA ratio	0.02 (-0.08; 0.12)	0.701	0.002 (-0.10; 0.11)	0.974	-0.04 (-0.14; 0.06)	0.403
Linoleic acid	-0.03 (-0.13; 0.08)	0.632	0.01 (-0.10; 0.11)	0.904	0.04 (-0.06; 0.15)	0.404
Saturated FA	-0.01 (-0.11; 0.09)	0.806	-0.01 (-0.11; 0.09)	0.856	0.04 (-0.06; 0.14)	0.458
Saturated FA ratio	0.12 (0.001; 0.24)	0.048	-0.11 (-0.23; 0.01)	0.085	-0.11 (-0.23; 0.01)	0.061
Monounsaturated FA	-0.04 (-0.14; 0.06)	0.402	0.01 (-0.09; 0.11)	0.817	0.07 (-0.03; 0.17)	0.151
**Metabolic substrates**						
Glucose	-0.11 (-0.22; -0.01)	0.030	0.14 (0.03; 0.24)	0.012	0.06 (-0.04; 0.17)	0.213
Glycerola	0.02 (-0.08; 0.13)	0.659	0.005 (-0.10; 0.11)	0.932	-0.05 (-0.15; 0.05)	0.306
Acetoacetatea	-0.04 (-0.15; 0.08)	0.530	0.04 (-0.08; 0.16)	0.514	0.03 (-0.09; 0.14)	0.647
ǂ 3-hydroxyburate	0.11 (0.03; 0.20)	0.009	-0.10 (-0.19; -0.02)	0.019	-0.12 (-0.20; -0.04)	0.003
ǂ Acetate	0.20 (0.09; 0.30)	<0.001 *	-0.18 (-0.29; -0.07)	0.001 *	-0.17 (-0.28; -0.06)	0.002 *
Citrate	-0.001 (-0.12; 0.12)	0.992	-0.02 (-0.14; 0.10)	0.725	0.03 (-0.09; 0.14)	0.635
Lactatea	0.02 (-0.09; 0.12)	0.740	-0.05 (-0.15; 0.06)	0.384	0.02 (-0.08; 0.12)	0.663
Pyruvate	0.02 (-0.09; 0.12)	0.740	0.03 (-0.08; 0.13)	0.593	-0.07 (-0.17; 0.03)	0.168
**Amino acids**						
Isoleucine (BCAA)	0.003 (-0.11; 0.11)	0.950	-0.02 (-0.14; 0.09)	0.687	0.02 (-0.09; 0.13)	0.696
Leucine (BCAA)	0.04 (-0.07; 0.15)	0.522	-0.05 (-0.16; 0.06)	0.345	-0.01 (-0.11; 0.10)	0.915
Valine	0.11 (-0.002; 0.22)	0.055	-0.11 (-0.23; 0.002)	0.054	-0.08 (-0.19; 0.03)	0.138
Alanine	-0.07 (-0.18; 0.04)	0.231	0.03 (-0.08; 0.14)	0.634	0.10 (-0.01; 0.21)	0.062
Glutamine	-0.13 (-0.22; -0.03)	0.012	0.15 (0.05; 0.25)	0.004	0.07 (-0.02; 0.17)	0.145
Glycinea	-0.04 (-0.15; 0.07)	0.451	0.05 (-0.06; 0.17)	0.374	0.02 (-0.09; 0.13)	0.680

Histidine	-0.01 (-0.12; 0.11)	0.932	-0.03 (-0.15; 0.09)	0.626	0.05 (-0.07; 0.16)	0.420
Phenylalaninea	-0.07 (-0.18; 0.03)	0.151	0.05 (-0.05; 0.16)	0.309	0.08 (-0.02; 0.18)	0.097
Tyrosine	-0.17 (-0.28; -0.06)	0.003	0.15 (0.04; 0.26)	0.008	0.15 (0.04; 0.26)	0.006
**Miscellanous**						
Glycoproteins	-0.06 (-0.16; 0.03)	0.190	0.07 (-0.03; 0.16)	0.184	0.05 (-0.05; 0.14)	0.319
Creatinine	0.07 (-0.05; 0.18)	0.274	-0.09 (-0.20; 0.03)	0.159	-0.03 (-0.14; 0.09)	0.656
Albumin	0.02 (-0.08; 0.12)	0.710	-0.05 (-0.16; 0.05)	0.308	0.03 (-0.07; 0.13)	0.585

ǂ Quantile regression models applied,* $p \leq 0.002$.

Table S4: Standardized single regression coefficients (β) of sedentary time and physical activity intensities with their 95% confidence intervals (CI) from age, education, smoking, alcohol, nutrition and aerobic fitness-adjusted linear regression models for serum metabolome.

**Metabolome measure**	**Sedentary time**		**Light intensity physical activity**		**Moderate-to-vigorous physical activity**	
	**β (95% CI)**	**p-value**	**β (95% CI)**	**p-value**	**β (95% CI)**	**p-value**
**Lipoprotein particle concentration**						
ǂExtremely large VLDL	-0.02 (-0.11; 0.06)	0.597	0.005 (-0.08; 0.09)	0.912	0.06 (-0.03; 0.14)	0.193
ǂVery large VLDL	-0.01 (-0.12; 0.10)	0.844	0.01 (-0.10; 0.11)	0.898	0.06 (-0.05; 0.17)	0.282
ǂLarge VLDL	0.004 (-0.12; 0.12)	0.952	-0.01 (-0.13; 0.10)	0.803	0.05 (-0.06; 0.17)	0.347
ǂMedium VLDL	-0.01 (-0.13; 0.11)	0.831	-0.02 (-0.14; 0.10)	0.733	0.03 (-0.08; 0.15)	0.577
Small VLDL	-0.03 (-0.13; 0.06)	0.506	0.00 (-0.10; 0.10)	0.999	0.07 (-0.03; 0.16)	0.162
Very small VLDL	0.03 (-0.08; 0.13)	0.626	-0.03 (-0.13; 0.07)	0.531	-0.01 (-0.11; 0.09)	0.848
IDL	0.03 (-0.07; 0.14)	0.528	-0.03 (-0.13; 0.08)	0.624	-0.04 (-0.14; 0.07)	0.501
Large LDL	0.03 (-0.08; 0.14)	0.604	-0.02 (-0.13; 0.09)	0.703	-0.03 (-0.14; 0.07)	0.560
Medium LDL	0.02 (-0.08; 0.13)	0.665	-0.02 (-0.13; 0.09)	0.738	-0.02 (-0.13; 0.08)	0.642
Small LDL	0.02 (-0.09; 0.13)	0.713	-0.02 (-0.13; 0.09)	0.729	-0.02 (-0.12; 0.09)	0.755
Very large HDL	0.03 (-0.08; 0.14)	0.595	-0.04 (-0.15; 0.07)	0.524	-0.01 (-0.12; 0.09)	0.787
Large HDL	0.01 (-0.09; 0.12)	0.798	-0.02 (-0.13; 0.08)	0.679	0.001 (-0.10; 0.10)	0.991
Medium HDL	-0.03 (-0.14; 0.09)	0.666	0.01 (-0.10; 0.13)	0.810	0.03 (-0.08; 0.15)	0.557
Small HDL	-0.03 (-0.15; 0.09)	0.603	0.02 (-0.09; 0.14)	0.701	0.03 (-0.08; 0.15)	0.560
**Lipoprotein particle size**						
VLDL diameter	-0.07 (-0.18; 0.04)	0.236	0.03 (-0.09; 0.14)	0.625	0.10 (-0.01; 0.22)	0.066
LDL diameter	0.02 (-0.10; 0.13)	0.775	0.02 (-0.10; 0.14)	0.741	-0.06 (-0.18; 0.05)	0.299
HDL diameter	0.03 (-0.08; 0.13)	0.618	-0.03 (-0.13; 0.08)	0.587	-0.02 (-0.12; 0.09)	0.744
**Apolipoproteins**						
ApoA1	-0.02 (-0.13; 0.10)	0.784	-0.01 (-0.12; 0.11)	0.902	0.04 (-0.07; 0.16)	0.460

ApoB	-0.01 (-0.11; 0.10)	0.884	-0.10 (-0.11; 0.09)	0.856	0.03 (-0.07; 0.13)	0.585
ApoB ApoA1 ratio	-0.002 (-0.10; 0.10)	0.973	-0.01 (-0.11; 0.09)	0.870	0.01 (-0.08; 0.11)	0.774
**Triglycerides**						
ǂTotal triglycerides	0.01 (-0.12; 0.13)	0.914	-0.04 (-0.16; 0.08)	0.510	0.03 (-0.09; 0.15)	0.662
ǂVLDL TG	-0.004 (-0.12; 0.12)	0.946	-0.05 (-0.17; 0.07)	0.424	0.05 (-0.07; 0.16)	0.442
IDL TG	0.09 (-0.003; 0.19)	0.057	-0.09 (-0.18; 0.01)	0.066	-0.07 (-0.17; 0.02)	0.121
LDL TG	0.08 (-0.02; 0.17)	0.113	-0.07 (-0.17; 0.02)	0.128	-0.06 (-0.16; 0.03)	0.195
HDL TG	-0.05 (-0.15; 0.05)	0.347	0.01 (-0.09; 0.11)	0.870	0.09 (-0.01; 0.18)	0.078
**Cholesterol**						
Total cholesterol	0.01 (-0.10; 0.12)	0.794	-0.02 (-0.13; 0.09)	0.741	-0.01 (-0.11; 0.10)	0.915
VLDL-C	-0.04 (-0.14; 0.07)	0.498	0.004 (-0.10; 0.11)	0.943	0.07 (-0.03; 0.17)	0.184
IDL-C	0.03 (-0.08; 0.14)	0.600	-0.02 (-0.13; 0.09)	0.715	-0.03 (-0.14; 0.07)	0.538
LDL-C	0.03 (-0.08; 0.13)	0.645	-0.02 (-0.13; 0.09)	0.760	-0.03 (-0.14; 0.08)	0.575
HDL-C	0.02 (-0.10; 0.13)	0.784	-0.02 (-0.13; 0.09)	0.719	-0.01 (-0.11; 0.10)	0.922
HDL2-C	0.01 (-0.10; 0.12)	0.819	-0.02 (-0.13; 0.09)	0.770	-0.01 (-0.11; 0.10)	0.925
HDL3-C	0.03 (-0.08; 0.15)	0.570	-0.05 (-0.16; 0.07)	0.409	-0.01 (-0.12; 0.11)	0.922
Esterified cholesterol	0.01 (-0.10; 0.12)	0.800	-0.02 (-0.13; 0.09)	0.754	-0.01 (-0.12; 0.10)	0.909
Free cholesterol	0.02 (-0.09; 0.12)	0.783	-0.02 (-0.13; 0.09)	0.711	-0.005 (-0.11; 0.10)	0.931
**Fatty acids**						
Total fatty acids	-0.03 (-0.13; 0.08)	0.601	-0.01 (-0.11; 0.10)	0.898	0.07 (-0.04; 0.17)	0.205
Unsaturation degree	0.01 (-0.10; 0.11)	0.911	0.02 (-0.08; 0.13)	0.674	-0.04 (-0.16; 0.08)	0.510
Omega-3 FA	-0.04 (-0.15; 0.07)	0.451	0.02 (-0.09; 0.13)	0.698	0.06 (-0.05; 0.17)	0.286
Omega-3 FA ratio	-0.04 (-0.15; 0.07)	0.484	0.04 (-0.07; 0.16)	0.441	0.03 (-0.09; 0.14)	0.655
Docosahexaenoic acid	0.001 (-0.11; 0.11)	0.987	-0.02 (-0.13; 0.09)	0.736	0.02 (-0.09; 0.13)	0.680
Polyunsaturated FA	-0.01 (-0.12; 0.09)	0.770	-0.01 (-0.12; 0.10)	0.910	0.04 (-0.07; 0.15)	0.445
Omega-6 FA	-0.01 (-0.12; 0.10)	0.840	-0.01 (-0.12; 0.10)	0.841	0.04 (-0.07; 0.15)	0.490
Omega-6 FA ratio	0.04 (-0.06; 0.15)	0.429	-0.003 (-0.11; 0.10)	0.958	-0.09 (-0.19; 0.02)	0.112
Linoleic acid	-0.02 (-0.13; 0.09)	0.785	-0.01 (-0.12; 0.10)	0.861	0.04 (-0.06; 0.15)	0.421
Saturated FA	-0.02 (-0.12; 0.09)	0.773	-0.02 (-0.12; 0.09)	0.739	0.06 (-0.05; 0.16)	0.295
Saturated FA ratio	0.10 (-0.02; 0.22)	0.107	-0.09 (-0.21; 0.03)	0.150	-0.09 (-0.21; 0.03)	0.143
Monounsaturated FA	-0.05 (-0.16; 0.06)	0.357	0.004 (-0.10; 0.11)	0.938	0.10 (-0.01; 0.20)	0.067
**Metabolic substrates**						
Glucose	-0.13 (-0.23; -0.02)	0.019	0.14 (0.03; 0.25)	0.010	0.08 (-0.03; 0.18)	0.138
Glycerola	0.01 (-0.10; 0.12)	0.871	0.005 (-0.10; 0.11)	0.929	-0.02 (-0.13; 0.08)	0.647
Acetoacetatea	-0.03 (-0.15; 0.09)	0.584	0.03 (-0.08; 0.15)	0.571	0.02 (-0.09; 0.14)	0.692
ǂ3-hydroxybuturate	0.11 (0.03; 0.19)	0.008	-0.10 (-0.19; -0.01)	0.026	-0.11 (-0.19; -0.04)	0.005
ǂAcetate	0.15 (0.03; 0.27)	0.012	-0.16 (-0.27; -0.05)	0.006	-0.14 (-0.25; -0.03)	0.013
Citrate	0.01 (-0.11; 0.13)	0.863	-0.03 (-0.15; 0.09)	0.624	0.02 (-0.10; 0.14)	0.773

Lactatea	0.01 (-0.09; 0.12)	0.816	-0.05 (-0.16; 0.06)	0.355	0.04 (-0.07; 0.14)	0.460
Pyruvate	-0.01 (-0.11; 0.10)	0.890	0.04 (-0.06; 0.15)	0.404	-0.04 (-0.15; 0.06)	0.415
**Amino acids**						
Isoleucine (BCAA)	-0.01 (-0.13; 0.10)	0.822	-0.02 (-0.14; 0.09)	0.699	0.06 (-0.06; 0.17)	0.325
Leucine (BCAA)	0.02 (-0.09; 0.13)	0.744	-0.05 (-0.17; 0.06)	0.371	0.03 (-0.08; 0.14)	0.617
Valine	0.10 (-0.01; 0.21)	0.088	-0.11 (-0.22; 0.005)	0.060	-0.06 (-0.18; 0.05)	0.276
Alanine	-0.05 (-0.17; 0.06)	0.386	0.004 (-0.11; 0.12)	0.942	0.10 (-0.01; 0.22)	0.085
Glutamine	-0.11 (-0.21; -0.01)	0.036	0.14 (0.04; 0.24)	0.007	0.04 (-0.06; 0.14)	0.421
Glycinea	-0.03 (-0.14; 0.09)	0.665	0.04 (-0.07; 0.16)	0.485	-0.001 (-0.11; 0.11)	0.985
Histidine	0.002 (-0.12; 0.12)	0.978	-0.04 (-0.16; 0.08)	0.524	0.05 (-0.07; 0.17)	0.432
Phenylalaninea	-0.08 (-0.19; 0.04)	0.184	0.04 (-0.07; 0.15)	0.489	0.10 (-0.01; 0.21)	0.062
Tyrosine	-0.16 (-0.28; -0.05)	0.006	0.13 (0.02; 0.25)	0.024	0.16 (0.05; 0.28)	0.005
**Miscellanous**						
Glycoproteins	-0.09 (-0.19; 0.01)	0.082	0.07 (-0.03; 0.17)	0.177	0.09 (-0.01; 0.19)	0.063
Creatinine	0.06 (-0.06; 0.17)	0.351	-0.08 (-0.20; 0.04)	0.195	-0.01 (-0.13; 0.10)	0.810
Albumin	0.03 (-0.07; 0.14)	0.563	-0.06 (-0.17; 0.04)	0.241	0.02 (-0.09; 0.12)	0.736

‡ Quantile regression models applied, * $p \leq 0.002$.
